# Advantages of the Cartilage-Sparing Technique in Prominent Ear Deformity Surgery: Comparison of a New Technique

**DOI:** 10.7759/cureus.43545

**Published:** 2023-08-15

**Authors:** Oguz Guvenmez, Anara Keneshovna Zhanbaeva, Huseyin Keskin, Adylbek Zhanbaev

**Affiliations:** 1 Otorhinolaryngology, Osh state University, Osh, KGZ; 2 Clinical Pharmacology, Osh State University, Osh, KGZ; 3 Otorhinolaryngology, Private Mersin Su Hospital, Mersin, TUR; 4 General Surgery, Osh State University, Osh, KGZ

**Keywords:** new technique in otoplasty, otoplasty, cartilage-protective method, traditional cartilage-removal technique, ear cartilage, surgical intervention, prominent ear

## Abstract

Objective: Ear cartilage, crucial for maintaining ear shape and function, can sometimes undergo damage or deformation, requiring surgical intervention. This study aimed to compare the efficacy of a novel, less invasive cartilage-protective method with the traditional, more invasive cartilage-removal technique.

Methods: Our study included 64 patients (128 ears). The first group of 32 patients (64 ears) received the new cartilage-protective technique, while the second group of an equal number of patients and ears underwent the traditional method. The newer technique endeavors to retain as much healthy cartilage as possible, addressing only the issue at hand, while the traditional technique requires the removal of a substantial portion of cartilage.

Results: The cartilage-protective method demonstrated several notable advantages over the traditional one. First, it significantly reduced the operation duration due to its less invasive nature. Second, it caused less pain to the patients by minimizing trauma to surrounding tissues. Furthermore, this technique significantly lowered the risk of complications, probably due to the minimal disturbance or removal of healthy cartilage, hence reducing the likelihood of post-operative complications such as infections or deformities.

Conclusion: The findings of our study propose the cartilage-protective method as a superior treatment option when surgical intervention becomes necessary to repair or restore the function of ear cartilage. This technique, being less invasive, not only results in less pain for the patients but also reduces the risk of complications. It promotes quicker patient recovery without any loss of sensation in the ear. Thus, it could potentially revolutionize the approach to dealing with ear cartilage issues.

Level of evidence: Level four

## Introduction

The auricle protruding from the side of the head develops from mesenchymal proliferations in the first and second pharyngeal arches (ear crests) surrounding the first pharyngeal groove [1]. Prominent ear is the most common pinna deformity. It is usually caused by the looseness of the cartilages in the ear region, the ear cartilages being out of place, and insufficient ear folds. For these reasons, the ear, which has a loose structure, bends forward or to the side, causing an unpleasant appearance [2]. Prominent ear is an auricular anomaly that is seen in the average 5% of the population and develops differently from the anatomical features that the auricle should normally develop. The incidence of the prominent ear in the community is 5%, and it may cause psychosocial diseases in affected individuals [3]. This pathology has many causes. It may be due to genetic mutations, familial transmission, or lying position disorders as an infant [4]. This ear anomaly, insufficient maturation of the antihelix, anterior rotation of the concha, hypertrophic concha, deep concha, >90 increase in the conchoscapal angle, or one or more of these anomalies may be present [5]. This problem causes children to not go out or style their hair as they want, shuts them down, and deteriorates their psychology. Although the physiological consequences are insignificant, the psychological and aesthetic consequences for the patient can be considerable [6]. The ear cartilage does not fully develop in children up to six years of age [7-8].

To date, a variety of techniques have been employed in otoplasty procedures. Cutting and removing is the most common cartilage technique from the very beginning to the present day; however, it is a technique made by throwing stitches. However, this technique has been found to have many disadvantages. Complications such as hematoma, cartilage necrosis, seroma, necrosis, severe pain, and numbness are seen. Otoplasty has started to be preferred more and more at a time when aesthetic operations are increasing frequently.

Various techniques have been used so far. The cartilage-sparing technique's advantages have been demonstrated in previous studies [9]. The aim of this study is to reveal the results and advantages of our cartilage-sparing technique in patients.

This case-control study aimed to compare the effectiveness of a new cartilage-sparing otoplasty technique with the traditional cartilage removal method in the surgical correction of prominent ears.

## Materials and methods

The files of patients who underwent primer prominent ear surgery by the same surgeon between October 1, 2021, and October 1, 2022, at Private Mersin Su Hospital, Mersin, Turkey, were retrospectively reviewed. Written informed consent was obtained from the patients that the results of the surgery would be used for scientific purposes by keeping their personal information confidential, and local ethics committee approval was obtained from the Mersin University Ethics Committee before starting the study (approval number: 07.12.2022/798).

The innovative otoplasty technique involves the use of a suture to reshape the ear without the need to remove any cartilage. A total of 32 patients (64 ears) received the new technique (Group 1), while 32 patients (64 ears) received the traditional method (Group 2).

The selection of surgical technique was randomized, ensuring a fair comparison between the two methods. A computer-generated randomization sequence, stratified by sex and age, was used to assign the 64 patients to either Group 1 (the new technique) or Group 2 (the traditional method).

Patients with congenital or acquired ear anomalies besides prominent ears were excluded. Before, during, and three months after the surgery, front, back, and side photographs of all patients were taken. Patients who received the new technique had conchal hernias and an unfurled antihelix. The results of the study will be compared to evaluate the effectiveness of the new technique in achieving aesthetically pleasing and long-lasting results.

Before the surgery, routine ENT examinations and the anatomical structures of the auricle were conducted by an otolaryngologist, who used this information to make appropriate pre-operative plans. This ensured that the team had a thorough understanding of the patient's ear anatomy and any potential surgical challenges, allowing them to tailor the surgical approach to the individual needs of each patient. The surgery was performed under general anesthesia, and patients were asked to rate their post-operative pain using a visual pain scale. This allowed the team to monitor the patients' pain levels and ensure that they were comfortable during the post-operative period.

After the surgery, daily dressings were applied for a period of five days. In this technique, no head wrapping is done. Instead, the surgical site is simply covered with light gauze and an adhesive strip. Patients were routinely followed up at the third and sixth months post-operatively to monitor their recovery and assess the results of the surgery. During the six-month follow-up, patients were asked to evaluate the results of their prominent ear surgery using a five-point scale ranging from "very bad" to "very good." This allowed the team to assess the overall satisfaction of the patients with the surgery and determine if the desired aesthetic outcomes were achieved. In addition, both patient and surgeon satisfaction was assessed using a five-point visual analog scale, with a score of one indicating "excellent" and a score of five indicating "poor." This enabled the team to gauge the overall satisfaction of both the patient and the surgeon with the surgical process and the outcomes.

On the other hand, both groups of patients were asked to score pain from one to 10 using the visual analog scale after the operation.

To further assess the outcomes of the surgery, the length and width of the ear and the upper, middle, and lower cephaloauricular distances were also measured and compared before and after the surgery. These measurements allowed the team to quantify any changes in the shape and size of the ears and determine if the surgery resulted in any functional improvements.

All early complications, such as hematoma, seroma, wound dehiscence, infection, and skin or cartilage necrosis, were recorded, and adverse conditions such as asymmetry, helix behind the antihelix, overcorrection, undercorrection, telephone deformity, protruding upper pole, protruding earlobe, non-round antihelix, upper shell, and other cartilage irregularities were also noted and compared statistically.

Surgical procedure

Preparations for the surgery began half an hour prior, during which time the patient was administered 500 mg of cefazolin as a prophylactic measure against infection. Once the patient had received general anesthesia, the surgery could commence.

The ear to be operated on was carefully positioned, and a sterile drape was applied to ensure a clean environment. Using a surgical pen, elliptical lines were drawn on the skin to mark the incision line, which ran from the point where the auricle attaches to the scalp to the postauricular sulcus.

Next, a mixture of 3 cc of 2% lidocaine and 1:100,000 epinephrine was diluted with physiological saline at a ratio of 1/2. This solution was then injected gently using a 30-gauge needle in order to control bleeding and perform hydrodissection. The skin and subcutaneous fat tissues were elevated from the top to the bottom to reach the perichondrium, and an opening approximately 4-5 cm in size was created from the periosteum on the mastoid bone to the posterior wall of the external auditory canal. A 3/0 polydioxanone (PDS) suture was used to complete the suture.

The periosteum was sutured first, followed by the reciprocal matrix being sutured over the eminentia fossa triangularis. Matrix stitches were placed at intervals of 1 cm towards the inferior, with four or five parallel lines being thrown in this plane. The concha mastoid distance was reduced by loosening the sutures and then tightening them all together. The suture was then cut without knotting, leaving a residue so that the knots could be tightened later. Finally, the knots were tightened one by one, bringing the auricle closer to the mastoid.

Overall, the surgeries were successful, resulting in the ear being repositioned to a more aesthetically pleasing position. The patient can now anticipate an enhanced appearance.

Statistical analysis

Once the data had been collected, they were analyzed using IBM SPSS Statistics version 21.0 (IBM Corp., Armonk, NY, USA). Normally distributed variables were summarized as the mean ± standard deviation, while nominal variables were presented as N (percentage). In order to compare the left and right ears before and after surgery, the paired sample t-test was used. A p-value of less than 0.05 was considered to be statistically significant, indicating that any changes that were observed were likely not due to chance alone.

The power analysis used in this study employed a margin of error of 5%, a power of 80%, and a standard effect size of 0.7. These values were chosen based on the specific aims of the study and the characteristics of the population being studied.

After conducting the power analysis, it was determined that a sample size of 32 cases in each group would be sufficient to accurately measure any changes that occurred as a result of the surgery. This sample size ensured that the results of the study would be statistically significant and able to withstand scrutiny when compared to other research on the topic.

## Results

The mean age of the patients was 18.38 years, and the average age between the two groups was similar. Of the participants in the study, four (53.1%) were male and 30 (46.9%) were female. The sex distribution was similar between the groups. All the patients were of the same gender as their sex (Table 1).

**Table 1:** Demographic variables of the study participants

	Mean (SD)	Range
Age (years)	18.38 (9.1)	6-37
	N	%
Sex		
Male	34	53.1
Female	30	46.9

In Group 1, the cephaloauricular distance was compared before and after the right ear surgery. While the mean pre-operative distance was 27.56 mm, the mean post-operative distance was 12.67 mm. For the left ear in Group 1, while the mean pre-operative distance was 28.15 mm, the mean post-operative distance was 11.87 mm (Table 2).

**Table 2:** Comparison of the cephaloauricular distance (mm) (before/after surgery) in Group 1 patients

	Pre-operative Mean (SD)	Post-operative Mean (SD)	t	df	p
Right Ear	27.56(2.9)	12.67 (3.5)	23.86	31	0.023
Left Ear	28.15(2.7)	11.87 (3.7)	23.92	31	0.018

In Group 2, the cephaloauricular distance was compared before and after the right ear surgery. While the mean pre-operative distance was 28.32 mm, the mean post-operative distance was 14.15 mm. For the left ear in Group 1, while the mean pre-operative distance was 29.08 mm, the mean post-operative distance was 13.69 mm (Table 3).

**Table 3:** Comparison of the cephaloauricular distance (mm) (before/after surgery) in Group 2 patients

	Preoperative Mean (SD)	Postoperative Mean (SD)	t	df	p
Right	28.32(2.6)	14.15 (3.6)	22.53	29	0.004
Left	29.08(2.4)	13.69 (3.3)	23.16	29	0.008

When the right and left ear post-operative cephaloauricular distances were compared for Groups 1 and 2, it was seen that there was a statistically significant difference for both ears (p<0.01) (Table 4).

**Table 4:** Group 1 vs. 2: The comparison of the cephaloauricular distance (mm) (right and left ear after surgery)

	Post-operative Mean (SD) for Group 1	Post-operative Mean (SD) for Group 2	t	df	p
Right Ear	12.67 (3.5)	14.15 (3.6)	13.12	29	0.003
Left Ear	11.87 (3.7)	13.69 (3.3)	12.26	29	0.007

The satisfaction rate of our patients who were operated on for a prominent ear was significantly higher in both groups. However, the satisfaction rate in Group 1 was significantly higher than in Group 2. Independent coroners often have stricter requirements (p<0.05) (Table 5).

**Table 5:** Patients' rate of satisfaction

	Group 1					Group 2					p
Scale	1	2	3	4	5	1	2	3	4	5	
Surgeon	7 (21,8%)	11 (34,4%)	11 (34,4%)	1 (3,1%)	2 (6,3%)	2 (6,3%)	6 (18,8%)	8 (25%)	7 (21,8%)	9 (28,1%)	0.008
Patient	16 (50%)	10 (31,2%)	6 (18,8%)	-	-	9 (28,1%)	8 (25%)	6 (18,8%)	4 (12,5%)	5 (15,6%)	0.041

According to the results of visual pain scoring, in Group 1 patients, the mean score was 2.5; in Group 2 patients, the score was 5.5; and it was found that the pain score was statistically lower in favor of Group 1 (p<0.05).

**Figure 1:**
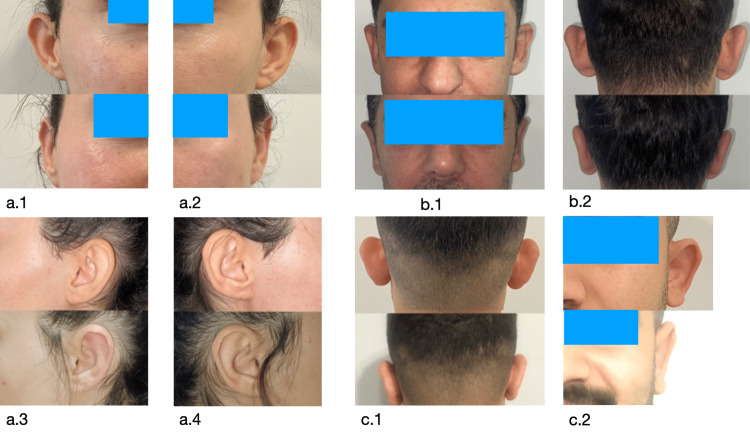
Pre- and post-operative comparative visual representation of the patients

The visual representation of the comparative results in patients pre-and post-operation can be seen in Figure 1.

**Figure 2:**
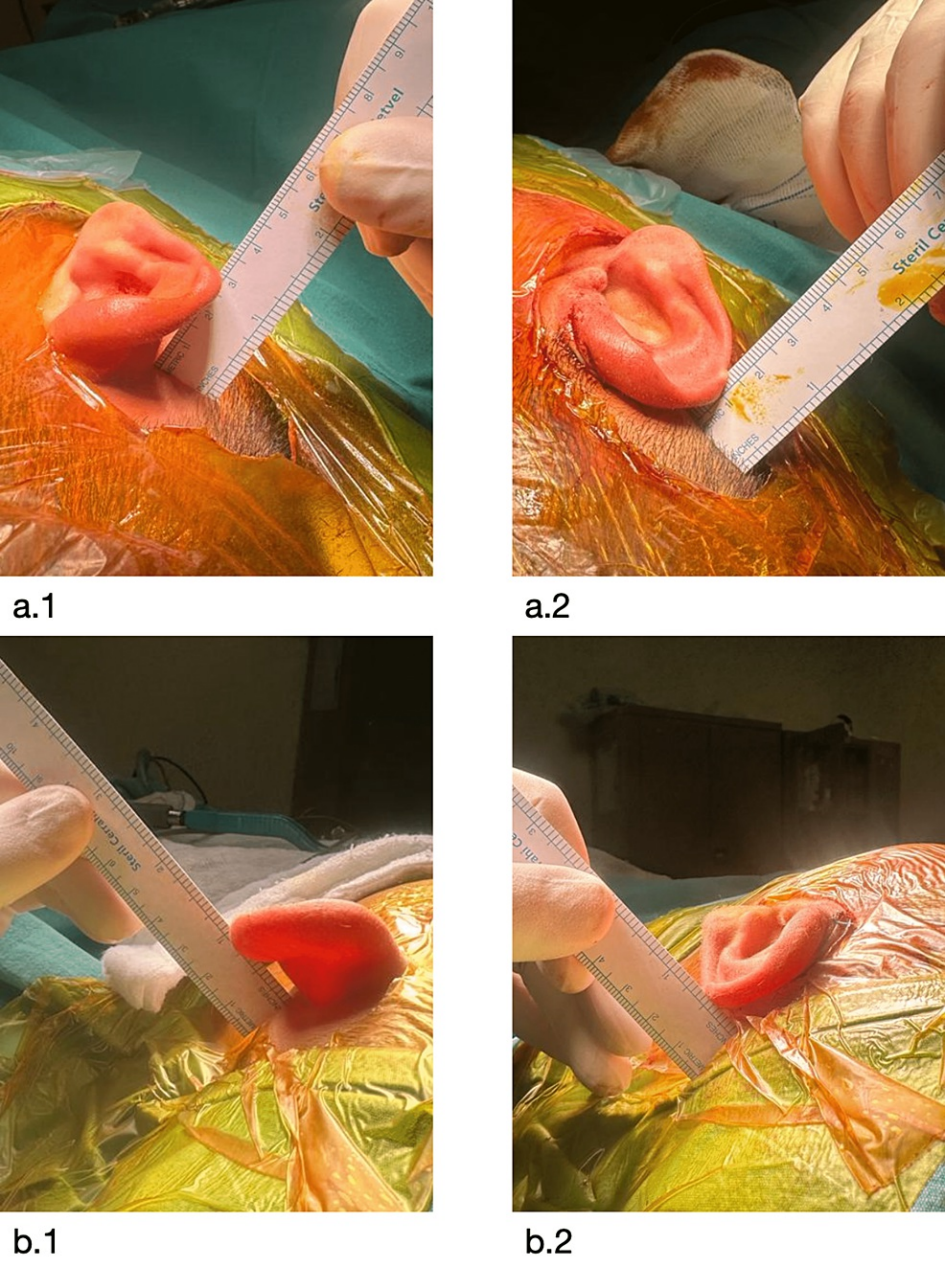
An intra-operative visual guide to the cartilage-sparing otoplasty technique

Intra-operative pictures of the technique are seen in Figure 2.

## Discussion

Otoplasty, also known as ear surgery or ear pinning, is a surgical procedure that is performed to improve the appearance of the ears [6]. This procedure can be done for a variety of reasons, including enhancing the aesthetic appeal of the ears, improving the symmetry and balance of the face, and addressing negative psychological effects such as low self-esteem or feelings of self-consciousness [10]. While the main goal of otoplasty is to improve the appearance of the ears, this procedure can also have significant psychological benefits that can positively impact an individual's quality of life [11].

Here, we want to introduce a new cartilage-sparing otoplasty technique. This means that the conchal cartilage is normal and protrudes out of its own bed. We called this an "ear hernia". This situation may occur with traumas or lying positions in the child's intrauterine life and childhood life.

One ear condition that can be addressed through otoplasty is an ear hernia. An ear hernia occurs when the conchal cartilage protrudes out of its own bed, resulting in a deformity of the ear. This condition can be caused by a variety of factors, including trauma or certain lying positions during fetal development. In some cases, a deep-pitted concha with a high posterior wall may also contribute to the prominence of the pinna and lead to the development of an ear hernia.

Traditionally, otoplasty techniques for addressing ear hernias have involved thinning or removing cartilage in order to correct the deformity. In addition, studies have shown that traditional cartilage removal techniques develop higher complications [12]. However, these approaches may result in additional scarring or a less natural appearance of the ear. In order to provide a more conservative and minimally invasive approach, we have developed a new cartilage-sparing otoplasty technique that aims to address ear hernias without thinning or removing cartilage.

This is a technique that we discovered for the first time. Thanks to this technique, there is less post-operative pain, and the surgeon does not have to worry about the development of complications. In the new technique that we applied to Group 1 patients, minimal auricular hematoma developed in only one patient out of 32. However, in Group 2, retroauricular hematoma occurred in two patients, minimal cartilage necrosis in one patient, skin incision in one patient, and excessive adhesion occurred in one patient.

Not only does this technique have the potential to improve the aesthetic appearance of the ears, it also has the potential to provide improved surgical outcomes and a faster recovery period for patients. In addition, the psychological benefits of otoplasty are well established, and this innovative approach has the potential to provide a more positive experience for individuals seeking to improve the appearance of their ears. Overall, we believe that this new cartilage-sparing otoplasty technique has the potential to be a valuable addition to the field of ear surgery and may benefit a wide range of individuals who are dissatisfied with the appearance of their ears. In addition, with the new technique we applied, there is no need to apply a bandage for one week post-operatively. Only sterile wound dressing and simple closure are sufficient.

In the clinical studies conducted to date, it has been observed that the cartilage-preserving technique is less painful, has shorter operation times [13], and has a lower complication rate compared to the cartilage-removing technique [14]. Cartilage-sparing techniques have continued to play a significant role in modern otoplasty, offering reduced risk of skin necrosis, cartilage irregularities, hematoma, and infection due to the non-invasive nature of the approach. However, refinements are often required to further decrease the incidence of recurrence and suture extrusion.

The deep-pitted concha with a high posterior wall is often an important factor in the prominence of the pinna; sometimes it's the only reason for the prominent ears.

Whatever evaluation system is used, such quality control studies involve a critical outcome analysis of postoperative complications, although subjective bias in the evaluation of cosmetic surgery outcomes can never be completely eliminated and such assessments are far from 'evidence-based'. Flaws and unsatisfactory results are quite helpful. Surgeons can accurately assess the quality of their work and examine the effects of developing new techniques. Using such studies, they can identify the causes of defects so that they can be corrected and prevented [15].

In our novel technique, there are significant differences from the Furnas approach. In the Furnas method, sutures are applied in a manner corresponding from the conchal cartilage to a point on the mastoid periosteum. However, in our technique, before placing the suture, a niche is created in the mastoid fossa where the conchal cartilage will be positioned. Following this, the conchal cartilage is slid into this niche using a specific sliding technique. The fixation sutures are initiated only after this procedure is completed. Initially, sutures are placed at the deepest point corresponding to the base of the fossa with a 3-0 Prolene. Subsequent sutures are applied parallelly from the conchal scaphal sulcus towards the mastoid periosteum.

Further research is needed to fully understand the potential benefits and drawbacks of the new cartilage-sparing technique. However, initial results suggest that it may offer a promising alternative to the traditional method. The basic principle of this surgery is to reduce the helix-mastoid distance without thinning or removing cartilage. This is achieved by placing the displaced scaphal cartilage into the mastoid pit using a sliding technique and by placing traction sutures from the scapha to the mastoid and fixing the scaphal cartilage to the mastoid pit with the help of the periosteum. Another way to reduce excessive turbinate cupping is to close the gap between the concha and mastoid processes. Overall, this study suggests that the new cartilage-sparing otoplasty technique is a safe and effective option for the surgical correction of prominent ears.

The limitation of this article is the low number of cases and the need for a longer follow-up period. Therefore, longer-term and larger-scale controlled clinical studies are required to address this.

## Conclusions

In this study, the cartilage-sparing technique we applied with the new technique has a shorter operation time and less pain than the cartilage-removal technique. In addition, the possibility of complications is very low, and the patient recovers quickly without loss of sensation in the ear. Long-term results and studies involving more patients are often needed with this new technique to decrease the incidence of recurrence and suture extrusion.
